# Effect of illumination on perceived temperature

**DOI:** 10.1371/journal.pone.0236321

**Published:** 2020-08-10

**Authors:** Yoshiaki Tsushima, Sho Okada, Yuka Kawai, Akio Sumita, Hiroshi Ando, Mitsunori Miki

**Affiliations:** 1 Center for Information and Neural Networks (CiNet), National Institute of Information and Communications Technology (NICT), Kyoto, Japan; 2 Faculty of Science and Engineering, Doshisha University, Kyoto, Japan; 3 Kimura Kohki Co. Ltd, Osaka, Japan; RMIT University, AUSTRALIA

## Abstract

The widely known hue-heat effect, a multisensory phenomenon between vision and thermal sensing, is a hypothesis based on the idea that light and colors affect perceived temperature. However, the application of this effect has not been prevalent in our daily lives. To work towards developing more practical use of the hue-heat effect, we conducted a series of psychophysical experiments to investigate the relationship between perceived temperature and illumination in a well-controlled experimental environment. The results showed that illumination had three types of effects to change our sense of coolness/warmness: creating, eliminating, and exchanging effects. Furthermore, we confirmed the existence of two distinctive time courses for the three effects: creating effect started immediately, but the eliminating effect takes time. These findings provide us with a better understanding of the hue-heat effect and enable us to apply it in everyday life. Paired with the new technologies it can also help with energy conservation.

## Introduction

Our sensory system often combines information from two or more sensory modalities, a process called multisensory integration. There are a large number of investigations on multisensory integration between audition-vision [1–3], vision-touch [4–6], auditory-touch [7,8], and others. Most academic research papers on multisensory integration have demonstrated multisensory phenomena by showing the influence of one sensory modality on the other. For instance, Shams et al. reported a multisensory phenomenon between sight and sound: a single visual flash is falsely perceived as multiple flashes when a single flash is accompanied by multiple auditory beep sounds [3]. However, applications that use such a multisensory illusion are less common in everyday life. The purpose of this study was to explore how we can use such phenomena more effectively in our daily lives.

Almost a century ago, psychologists reported that a sensation of coolness/warmness was influenced by perceived visual color, which is now called the hue-heat effect [9]. The hue-heat effect indicates that a cool ambient color creates a perception of cooler temperature and that a warm color leads to a warmer temperature perception. Needless to say, the hue-heat effect is one of the multisensory phenomena between vision and thermal sensing. There have been various researches about the hue-heat effect [10–19], and we are yet to accumulated scientific knowledge about it. For example, a recent study revealed that priming a color affected thermal discrimination [20]. In another example, Ho et al. suggested that prior expectations about temperature influenced the direct touch temperature [21]. However, most of the data obtained in these investigations are not used for developing our daily applications. One of the main reasons is that these data were not obtained in well-controlled conditions which included illumination, color temperature, and humidity. Also, some investigations and analyses fell short of the level needed for scholarly research in psychophysics, such as the lack of a control experiment for understanding the psychological phenomenon over time. Therefore, applications and systems with the hue-heat effect have not been developed for more practical situations yet.

To solve these problems, we conducted psychophysical experiments to rigorously investigate the hue-heat effect, especially the relationship between perceived temperature and illumination. Specifically, we rigidly controlled the experimental conditions, including illumination, color temperature, and humidity. Furthermore, we analyzed the subjective assessment over time to better understand the time course of the hue-heat effect. The obtained results will help us to design a real space using human multisensory integration as well as an advanced understanding of the psychological mechanism of the hue-heat effect.

## Materials and methods

### Participants

A total of 118 healthy people (66 males, 52 females) with a mean age of 21.43 years old participated in the experiments. All participants were recruited through the human recruitment bulletin board in Doshisha University in 2017 from August 6^th^ to 12^th^ and in 2018 from 21^st^ July to 27^th^. Some participants were excluded because they did not show up on time. Seventy participants were assigned to the main experiment, 15 for 0°C gap (males: 8, females: 7), 20 for 1°C gap (males: 14, females: 6), 13 for 2 °C gap (males: 8, females: 5), and 22 for 3°C gap groups (males: 12, females: 10). Forty-eight participants took the control experiment, 12 for each one of the four groups (0–3°C gap) (males: 6, females: 6 for each group). The post hoc analysis using PANGEA [22] indicated that these sample sizes and experimental designs (see the below) yielded 80% power to detect *d* = 0.45 [23]. All the participants provided written informed consent, and the research was approved by the Ethics Committee of Doshisha University, and it was in compliance with the Declaration of Helsinki. The individual in this manuscript has given written informed consent (as outlined in PLOS consent form) to publish these case details.

### Experimental period

All experiments were conducted in 2017 from August 12^th^ to 26^th^ and in 2018 from 27^th^ July to 16^th^ August in Japan. July and August are summer months in Japan. The average temperatures were 32.6°C high and 22.7°C low in 2017 and 35.4°C high and 24.5°C low in 2018. Average humidity was 65.5% in 2017 and 62.6% in 2018, in Kyoto.

### Facilities

**Fig 1:**
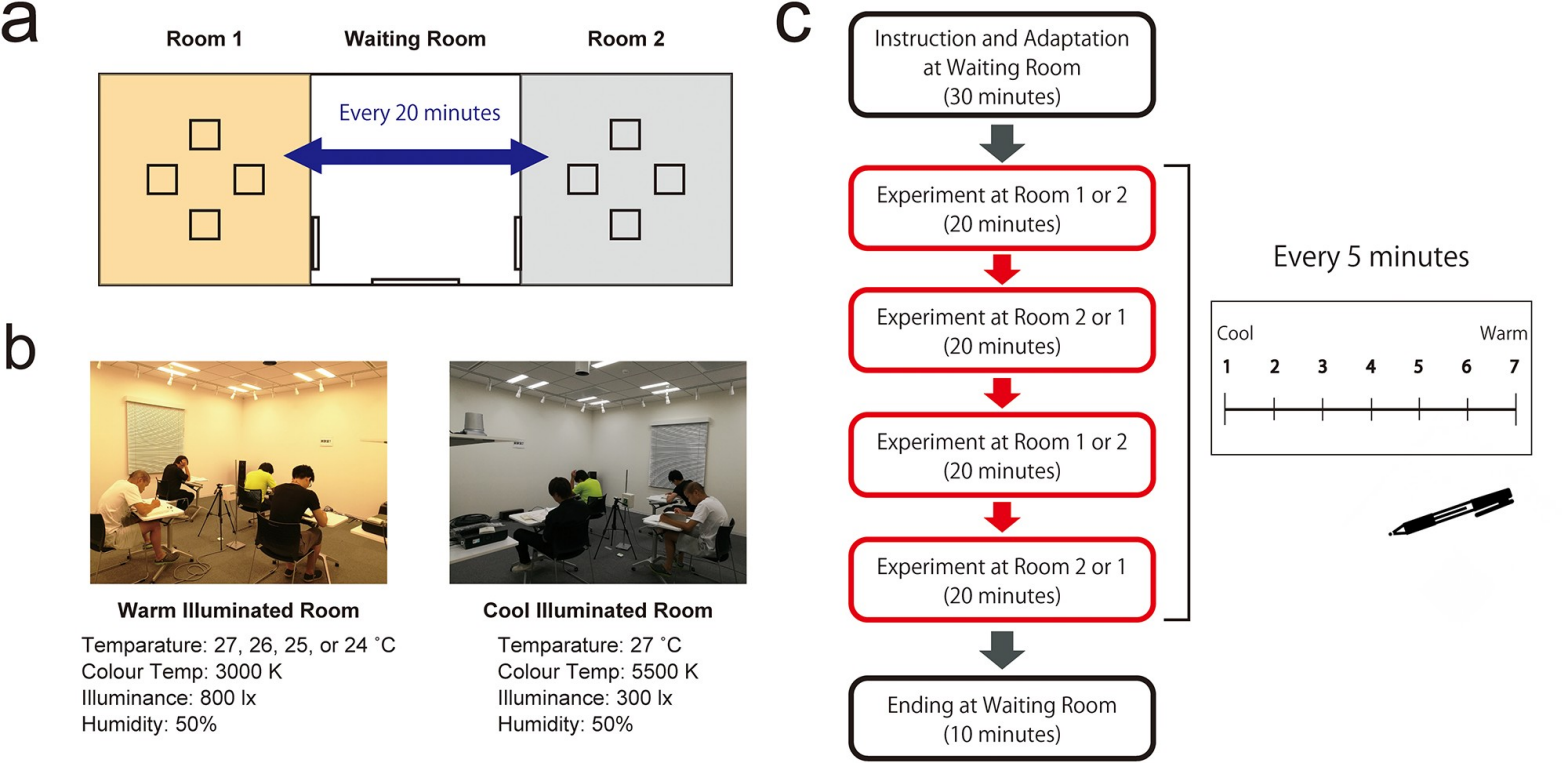
Design and procedure of the main experiment. (a) Experimental Rooms (Meta-comfort Lab, Kyoto, Japan): there were three rooms with no windows: two experimental rooms, and one waiting room. (b) Pictures of warm and cool illuminated rooms and environmental parameters of each experimental room. (c) Experimental design: participants entered and stayed in Room 1 or 2 for 20 minutes. In the room, they reported their sensation of coolness/warmness every 5 minutes. Then, they moved to Room 2 or 1, stayed for 20 minutes, and made the same report as last. The same experiment was conducted on them twice.

We used Meta-comfort Lab placed in Keihanna Open Innovation Center (Kyoto, Japan) for the experiment. There are three rooms: one waiting room and two experimental rooms (Fig 1a). The two experimental rooms had equal space (5.5 × 5.5 m^2^) and equipment and were made with the same materials. In both the experimental rooms, high-performance AHU with temperature and humidity control system, Air-Combi (KIMURA KOHKI Co. Ltd, Japan; S1 Fig), were installed. In addition, the ceiling had digital LED lights (DL-AG30VM, Sharp, Japan; S1 Fig). Both experimental rooms were designed according to the subject groups. For example, when testing the 2°C gap group, Room 1 or 2 was set at 25 °C, and Room 2 or 1 was set at 27 °C (Fig 1a and 1b). The degree of humidity in both experimental rooms was kept at 50% at all times (S2 Fig).

### Procedure

Participants were asked to wear the usual summer clothes in Japan: t-shirt and pants. First, participants listened to the experimental instructions in the waiting room and adapted to the experimental environment for almost 30 minutes (Fig 1a and 1c). Each participant was randomly assigned to one of the four participant groups: 0 degrees Celsius (°C) gap, 1 °C gap, 2°C gap, or 3°C gap group. The 0°C gap group meant that participants went back and forth between 27°C and 27°C rooms (27°C vs. 27°C). The 1 °C gap group was between 26°C and 27°C, the 2 °C gap group was between 25°C and 27°C, and the 3 °C gap group was between 24°C and 27°C. We set these different conditions based on 27°C as this is the air conditioning temperature during summer in Japan.

To ensure there wasn’t any bias, the participants were not told which experimental group they belonged to. To investigate the subjective assessment over time, groups of two to four participants entered and stayed at Room 1 or 2 for 20 minutes (Fig 1b). The room illuminated with warm color was set at a color temperature of 3000K and illuminance of 800lx. The room illuminated with cool color was set to a color temperature of 5500K and illuminance of 300lx. Both rooms were set to 50% humidity. In the experimental room, they were asked to spend time engaging in quiet activity (e.g. read a book) and report the sensation of perceived temperature every 5 minutes (0, 5, 10, 15, and 20 minutes later) on a seven-level scale, from cool to warm (Fig 1c).

After staying in Room 1 or 2 for 20 minutes, they were moved to Room 2 or 1, respectively. The continued to report the perceived temperature in the same manner. To check whether or not participants reported the consistent value of perceived temperature in the room, they performed the same task twice with intervals (Fig 1c). Hence, they reported the perceived temperature ten times for each room (Fig 1c).

As a control experiment, to check the accuracy of the subjective assessment of perceived temperature between two rooms, we conducted identical experiments with the exception that both experimental rooms had the same illumination and humidity but different temperatures. The values of the illumination parameters were almost the same in two rooms as in the main experiment: color temperature: 4500K, illuminance: 700lx, and humidity: 50%, and 24–27 °C.

### Data analysis

Each participant had two assessment values for each room and time elapsed. We calculated the mean value and used it as the assessment value of the room for each person.

## Results

**Fig 2:**
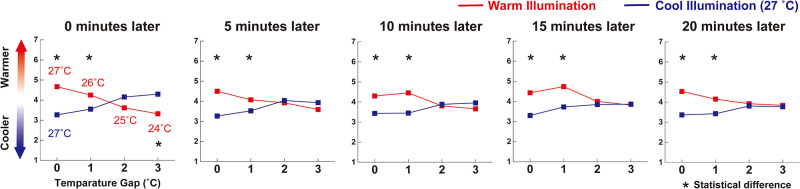
Results of mean subjective evaluations for perceived temperature in warm illuminated and cool illuminated rooms, at 0, 5, 10, 15, and 20 minutes. This clearly indicates that a sensation of coolness/warmness is manipulated by illumination.

The psychological data of the main experiment showed that a sensation of coolness/warmness was manipulated by illumination: participants felt cooler/warmer in a cool/ warm illuminated room. For one clear example, the participants felt difference in the degree of perceived temperature even at the same physical temperatures (see 27°C vs. 27°C in Fig 2). To expand more, it turned participants’ perceived temperature upside down at least 1°C gap from the moment participants entered the experimental rooms: participants felt cooler at 27°C than at 26°C at any time (see 26°C vs. 27°C in Fig 2; *p* < .05 at all conditions, t-test with Holm correction). Moreover, after five minutes, illumination could make participants feel no temperature difference even though there was a 2 or 3°C gap (see 25°C vs. 27°C and 24°C vs. 27°C in Fig 2). This indicates that such a sensory illusion seemed to be more effective over time.

**Fig 3:**
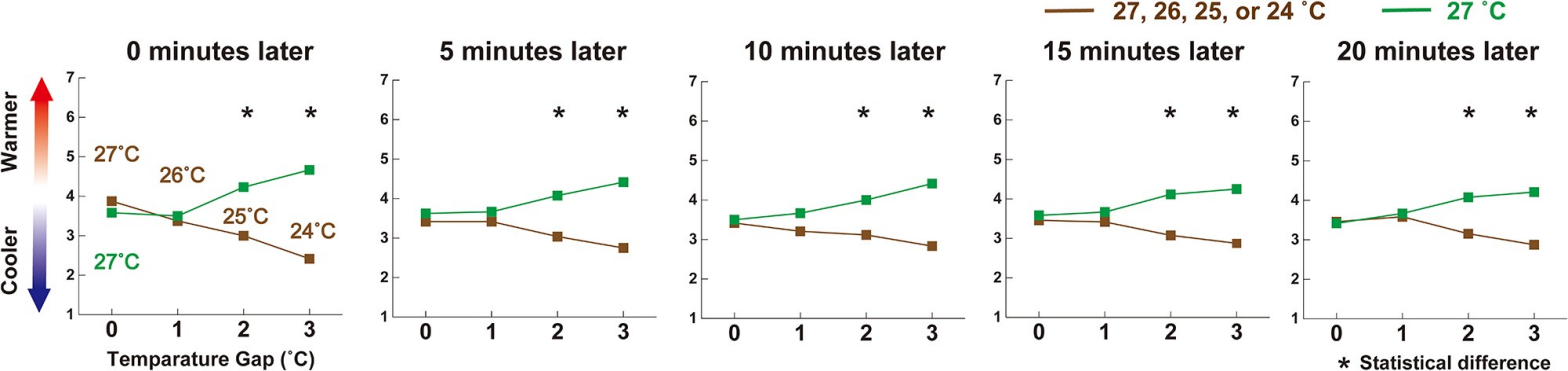
Results of mean subjective evaluations for perceived temperature in the same illuminated rooms at 0, 5, 10, 15, and 20 minutes. Participants noticed a 2 °C gap from the moment when they entered the room.

In the control experiment with the same illumination, the results showed that participants could report the different values of perceived temperature in the two experimental rooms (Fig 3).

In particular, from the moment they entered the participants could express the different values of perceived temperature from a 2°C gap f (Fig 3). This indicates that participants in the current study were able to detect a gap of at least 2 °C between the two experimental rooms.

To test the consistency of assessment in a different time, we compared their reports for a room at the first time with the second time (Fig 1c). As a result, we did not find any systematic differences between them in either the main or control experiments (see S3 Fig). This indicates that the order of effect over time did not contribute to our main finding.

## Discussion

**Fig 4:**
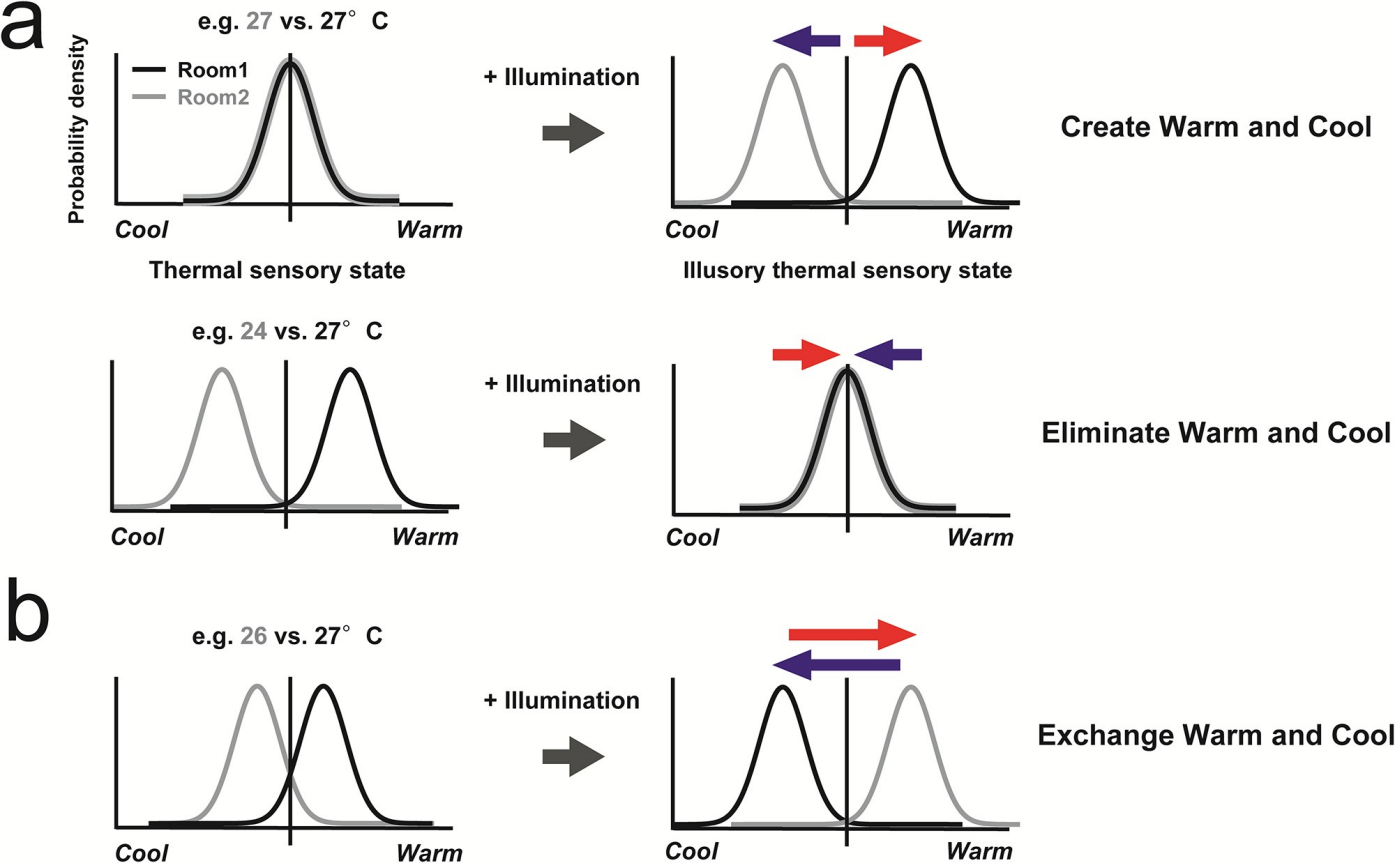
Thermal detection models of multisensory integration between perceived temperature and illumination (a) Top: Illumination creates warmness and coolness (See 27°C vs. 27°C condition in Fig 2). Bottom: Illumination eliminates warmness and coolness (See 25°C vs. 27°C or 24°C vs. 27°C in Fig 2). (b) Illumination exchanges warmness and coolness (see 26°C vs. 27°C in Figs 2 and 3).

The present results revealed that a sensation of coolness/warmness was manipulated by illumination. Specifically, there were two main effects: creating and eliminating effects. In the creating effect, illumination made participants feel different degrees of perceived temperature even at the same temperature condition (Top model in Fig 4a, see also 27°C vs. 27°C in Fig 2).

In the eliminating effect, illumination made the participants feel the same degree of perceived temperature under different temperature conditions (bottom model in Fig 4a; see also See 25°C vs. 27°C or 24°C vs. 27°C in Fig 3, and 25°C vs. 27°C or 24°C vs. 27°C after 5 min in Fig 2). In addition, this effect remained up to at least a 3°C gap in this study. Furthermore, the data indicates one more possible effect: the exchanging effect. The data showed that illumination made participants feel the degree of perceived temperature opposite to the actual temperature at 26°C vs. 27°C condition: they felt warmer in the room illuminated in warm color at 26°C than the room illuminated in cool color at 27°C (see 26°C vs. 27°C in Fig 2). Although our data did not represent different perceived temperatures in the same illuminated room between 26°C and 27°C, illumination might have the potential power of exchanging participants’ sensations of warmness and coolness (Fig 4b). These theoretical analyses for the relationship between perceived temperature and illumination contribute to building better knowledge of multisensory integration and perception in humans [24,25].

Additionally, investigation has revealed two things about the time course of the hue-heat effect, especially the relationship between perceived temperature and illumination. First, the effect was maintained for at least 20 minutes: participants reported the same or different degree of perceived temperature even at different or same physical temperature 20 minutes later (compare the graphs at 20 minutes later in Fig 2 with the one in Fig 3). Second, the effect took a small amount of time to begin in some situations, especially in the eliminating effect. For example, participants reported a different degree of perceived temperature between 24°C and 27°C at 0 minutes later (no effect), but they did report the same degree of perceived temperature at 5 to 20 minutes later (the eliminating effect). In contrast, it needed no time for the creating effect (see 27°C vs. 27°C at 0 minutes later in Fig 2). This indicates the presence of two distinctive time courses in the hue-heat effect. These analytical points of view about the time course might offer new insights into the hue-heat effect, which helps us to make daily applications and systems such as light-air fusion systems.

One might think that participants did not simply report their perceived temperature but made their assessment with excessively considering visual color temperature because some of them already had some knowledge of the hue-heat effect. However, this is unlikely. If that was the case, participants would consistently report warmer sensation in the room illuminated with warm color or made at least the same degree of evaluations between the two experimental rooms at any time. However, participants addressed the cooler in the warm illuminated room at 24°C vs. 27°C conditions at 0 minutes later (see Fig 2; 3 °C gap at 0 minutes later). This result cannot be directly attributed to this possibility. Therefore, we could trust that participants reported their perceived temperature without overreacting to the hue-heat effect. Thus, the current data would be psychophysically reliable and valuable evidence of the hue-heat effect between perceived temperature and illumination.

Moreover, these results contribute not only to the development of hue-heat for daily applications but also for the enhancement of energy conservation. According to a report by the Ministry of the Environment of Japan, turning up 2 °C of the air conditioning facility during summer approximately makes a 6.8% energy-saving effect [26]. Since the present study showed that the illumination possessed the power to eliminate at least 3 °C gap temperature (see the model in Fig 4a), the illumination would make no difference in perceived temperature even when changing the 2 °C setting up in summer. In other words, the illumination might make us feel more comfortable without relying on high energy-consuming air-conditioning systems. Therefore, the current data would be one of the rationales for national policy of energy conservation and human health.

In the present study, we conducted a series of psychophysical experiments during a specific period in a distinctive field in the summer of Japan. Although the obtained data are scientifically reliable, there are limitations to applying well to all seasons and situations in the world because our living spaces range widely. For example, it is expected that we have a slightly different outcome from the experiments during winter in Japan because of different humidity and air heating systems. Hence, we need to conduct similar experiments under various conditions to apply the present finding to a wide variety of situations and locations. Furthermore, we need to develop a theoretical understanding of the multisensory integration involving perceived temperature to find the more practical importance. A recent research review suggests that temperature-based correspondences consist of the framework of “statistical”, “structural”, “semantic”, and “affective” correspondences [27]. Such metaphysical and analytical approaches to the multisensory phenomenon of perceived temperature might make a new insight of the hue-heat effect. Although further research is needed to use the hue-heat effect in a variety of living situations, the current study definitely helps us to develop new technologies for our society as well as daily applications with the hue-heat effect.

## Supporting information

S1 Fig Pictures of MC-Lab. (DOCX)

S2 Fig Results of a humidity control experiment for 20 minutes. (DOCX)

S3 Fig Results of the mean assessment difference between 1^st^ and 2^nd^ time for each room. (DOCX)
