# Supplementary material for: Effect of illumination on perceived temperature
Source: PLoS One. 2020 Aug 10;15(8):e0236321. doi: 10.1371/journal.pone.0236321 (PMC7416916; doi:10.1371/journal.pone.0236321)
Supplement: S1 Fig — (DOCX) [file pone.0236321.s001.docx]

**
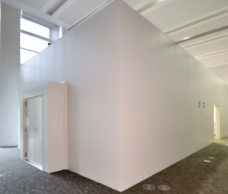

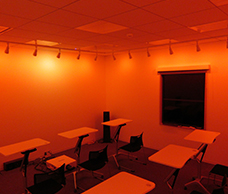
**

**S1 Fig. Pictures of MC-Lab.** Exterior view (left) and Interior view (right). MC-Lab is placed inside Keihanna Open Innovation Center building. It allows us to control the illumination and temperature with a fair amount of accuracy. There are no windows in the rooms (It looks a window, but it is a digital display that was not used in our experiment.)
