# Supplementary material for: Effect of illumination on perceived temperature
Source: PLoS One. 2020 Aug 10;15(8):e0236321. doi: 10.1371/journal.pone.0236321 (PMC7416916; doi:10.1371/journal.pone.0236321)
Supplement: S2 Fig — (DOCX) [file pone.0236321.s002.docx]

**S2 Fig.** **Results of a humidity control experiment for 20 minutes.** In this experiment, we tried to keep 50.0% humidity and 27 ℃ in the room. Averaged humidity was 48.7± 0.1% (SEM), and averaged temperature was 27.2± 0.02℃ (SEM) . This data represents that the system of MC-Lab possesses the power to control the humidity and temperature in a room.
