# Supplementary material for: Effect of illumination on perceived temperature
Source: PLoS One. 2020 Aug 10;15(8):e0236321. doi: 10.1371/journal.pone.0236321 (PMC7416916; doi:10.1371/journal.pone.0236321)
Supplement: S3 Fig — (DOCX) [file pone.0236321.s003.docx]

**S3 Fig.** **Results of the mean assessment difference between 1^st^ and 2^nd^ time for each room.** Asterisks indicate significant differences between the values and zero (t-test with Bonferroni correction). There were some small differences (all were less than absolute one-level scale.), however, we did not find any systematic tendencies (e.g. participants always reported that Room 1 was cooler/ warmer at 1^st^ time than 2^nd^ time.). Therefore, we concluded that the order effect over time did not significantly contribute to our main findings.
